# Adjunctive Therapies to Cerclage for the Prevention of Preterm Birth: A Systematic Review

**DOI:** 10.1155/2013/528158

**Published:** 2013-03-27

**Authors:** Emily A. DeFranco, Amy Miyoshi Valent, Tondra Newman, Jodi Regan, Jessica Smith, Louis J. Muglia

**Affiliations:** ^1^Maternal-Fetal Medicine, Department of Obstetrics and Gynecology, University of Cincinnati College of Medicine, Medical Sciences Building, Room 7212, 231 Albert Sabin Way, Cincinnati, OH 45267-0526, USA; ^2^Center for Prevention of Preterm Birth, Perinatal Institute, Cincinnati Children's Hospital Medical Center, Cincinnati, OH 45229-3026, USA

## Abstract

The aim of this paper is to provide a thorough summary of published studies that have assessed the efficacy of adjunctive therapies used in addition to cervical cerclage as a preventive measure for preterm birth. We limited our paper to patients treated with cerclage plus an additional prophylactic therapy compared to a reference group of women with cerclage alone. The specific adjunctive therapies included in this systematic review are progesterone, reinforcing or second cerclage placement, tocolytics, antibiotics, bedrest, and pessary. We searched PubMed and Cochrane databases without date criteria with restriction to English language and human studies and performed additional bibliographic review of selected articles and identified 305 total studies for review. Of those, only 12 studies compared the use of an adjunctive therapy with cerclage to a reference group of cerclage alone. None of the 12 were prospective randomized clinical trials. No comparative studies were identified addressing the issues of antibiotics, bedrest, or pessary as adjunctive treatments to cerclage. None of the 12 studies included in this paper demonstrated a clear benefit of any adjunctive therapy used in addition to cerclage over and above cerclage used alone; however, few studies with small numbers limited the strength of the conclusions.

## 1. Introduction

Cervical cerclage has been demonstrated to be an effective intervention in some subgroups of women who are at particularly high risk of spontaneous preterm birth or previable pregnancy loss. Because of the devastating outcomes associated with extreme preterm birth and previable pregnancy loss, obstetric care providers sometimes offer additional therapies in combination with cerclage that have shown benefit when used individually in other clinical scenarios for treatment of preterm labor and/or prevention of spontaneous onset of parturition. The purpose of this paper is to provide a summary of the existing medical evidence to support the use of adjunctive therapies or therapies used in addition to, cervical cerclage for the prevention of preterm birth by a systematic review of the currently available medical literature.

## 2. Data Sources and Study Selection

 For the purpose of this systematic review, we aimed to identify published studies that have assessed the efficacy of adjunctive therapies used in addition to standard prophylactic vaginal cervical cerclage as a preventive measure for preterm birth. We focused our paper to these specific adjunctive therapies that have been reported as used in addition to cerclage: progesterone, reinforcing (second) cerclage, tocolytics, antibiotics, bedrest, and pessary. We were primarily interested in studies assessing the efficacy of these adjunctive therapies with cerclage in routine or asymptomatic cases and therefore did not include studies that specified addition of the adjunctive therapy after cerclage because the patient subsequently became symptomatic, that is, the addition of tocolysis after a patient with cerclage developed symptomatic contractions or the addition of antibiotics in a patient with a previously placed cerclage who then developed signs or symptoms of intra amniotic infection. Because our focus was on additional therapies that may be used in routine practice in asymptomatic patients who undergo prophylactic cerclage placement, we referred to the 11 studies published in English language in the Cochrane review of randomized trials of cerclage in singleton pregnancies [1] to develop our definition of adjunctive therapy. We considered an adjunctive therapy as one that differed significantly from what has been usually and customarily incorporated as a therapy in addition to cerclage proximate to the time of cerclage placement in those trials included in the Cochrane review. One section of this systematic review, reinforcing cerclage does describe the use of an adjunctive therapy employed remote from the time of initial cerclage; however, we felt that this topic was pertinent to this paper as the majority of these patients were asymptomatic and had no clear new pregnancy complication other than progression of the original indication for preventative cerclage. Details of our definitions of adjunctive therapies are included in Section 3 that follows.

We searched PubMed and Cochrane databases without date criteria in September 2012 using the keywords “cerclage,” “repeat and cerclage,” “reinforcing and cerclage,” “revision and cerclage,” “progesterone and cerclage,” “atosiban and cerclage,” “beta mimetic and cerclage,” “terbutaline and cerclage,” “indomethacin and cerclage,” “magnesium and cerclage,” “nifedipine and cerclage,” “calcium channel blocker and cerclage,” “antibiotics and cerclage,” “bedrest and cerclage,” and “pessary and cerclage,” with restriction to English language and human studies. These search criteria identified 277 citations. A bibliographic review of selected articles was also performed to search for additional studies which may have been missed with the original search terms. Twenty-eight additional studies were identified, yielding 305 total studies for review. Review articles were excluded, as were articles on unrelated topics and those that did not report the use of therapies as an adjunct to a standard treatment of cerclage in comparison to a reference group. We also excluded studies that reported adjunctive therapies only in atypical scenarios of cerclage placement such as abdominal cerclage, vaginal cerclage placement following the delivery of a first born twin, or “emergent cerclage” placement after a patient presented with advanced cervical dilation and/or prolapsed membranes as an indication for the initial cerclage placement. Details regarding the number of articles remaining in each category of adjunct to cerclage are described individually in Section 3. Because of the paucity of published data regarding the use of various adjunctive therapies in addition to prophylactic cerclage placement, we chose to include a narrative description of some individual cases of descriptive studies such as case series with no reference group of women who received cerclage without adjunctive therapy. In these cases, we commented on the findings of the publication after specifically stating that there were no existing studies for that topic with a reference group for comparison.

## 3. Results 

### 3.1. Progesterone Therapy as an Adjunct to Cerclage

A literature search using the key words “progesterone and cerclage” identified 64 citations, and an additional 14 articles were detected using other sources. Articles were excluded from this paper for the following reasons: 47 were literature reviews or book chapters, 9 did not include a comparison group of patients who received cerclage plus progesterone therapy, 4 were case series of progesterone use with cerclage but did not include a reference group of cerclage without progesterone for comparison [8–10], 2 did not specify the duration of progesterone treatment, 1 reported technical failure of cerclage, 3 did not clearly report pregnancy outcomes, 4 were questionnaire/survey studies, and 2 included duplicate information on the same patient data published in more than one article. After exclusions, 6 published studies were selected for inclusion in this systematic review. 

 Cerclage placement in women with a history of spontaneous preterm birth and a shortened cervical length (<25 mm) has been shown to be beneficial for the prevention of preterm birth and adverse perinatal outcomes [11]; however, when used in women with no prior history of preterm birth, substantial improvement in maternal or neonatal outcomes has not been demonstrated [1]. Weekly intramuscular progestin therapy (17-alpha-hydroxyprogesterone acetate (17-*α*OHPC)) given to women with a singleton pregnancy and history of spontaneous preterm birth has also been shown to be effective in decreasing the incidence of preterm delivery [12], but few studies have focused on the combination of cerclage plus progesterone for the prevention of preterm birth (see Table 1). 

Our review identified 6 studies that compared the use of progesterone plus cerclage to a reference group of women who received cerclage without progesterone [2–7]. All 6 studies used the synthetic progestin 17-*α*OHPC, and none utilized vaginal progesterone or systemic administration of bioidentical progesterone. All of the studies included in this paper were observational cohort studies, 4 retrospective cohorts [4–7], 1 that was presented as a prospective cohort [3], and 1 observational study which was unable to be classified as prospective or retrospective in nature based on the review of the study design [2]. No clinical trials have yet been performed in which women who undergo cerclage are randomly allocated to receive 17-*α*OHPC versus placebo. The review of these 6 published studies does not suggest evidence to support that 17-*α*OHPC administration provides additional reduction in the incidence of recurrent preterm birth nor a synergistic benefit with cerclage when given preoperatively to cerclage placement or in any duration postoperatively until delivery [2, 3, 5–7]. The addition of 17-*α*OHPC to cerclage also was not associated with a reduced rate of previable births [4, 5]. Several of these studies reported insufficient sample size to identify a significant rate reduction in preterm birth if an effect of progesterone did exist [5–7]. Progesterone, however, does appear to be associated with a decrease in frequency of hospitalizations for preterm labor and uterine contractions, which may be attributable to progesterone's anti-inflammatory mechanisms, oxytocin inhibition, or improvement of immune function [4, 7]. Neither neonatal birth weight nor Apgar scores nor perinatal mortality were significantly influenced with the adjunct of progesterone therapy to cerclage, and other neonatal outcomes were not evaluated in any of the sources reviewed [4–6].

 Although currently available published studies have not demonstrated a cumulative improvement in the prevention of recurrent preterm birth with the combination of progesterone and cerclage, both therapies have been associated with a reduced incidence of preterm birth without increased maternal or neonatal morbidity [2, 3, 8–12]. Some data suggest that patients who receive progesterone therapy in addition to cerclage have fewer hospitalizations for preterm contractions, which could be beneficial to these high-risk patients both financially and psychologically. There is no available evidence from analytic studies to address the issue of whether other forms of progestins (vaginal progesterone or systemically administered bioidentical progesterone) may be beneficial when used as adjunctive therapy to cerclage, as all of the 6 published studies used 17-*α*OHPC. Before progesterone can be recommended as an adjunct to cerclage for standard cerclage indications, randomized clinical trials in which women who receive cerclage are randomized to progesterone versus placebo are needed with sufficient statistical power to determine if preterm birth and adverse perinatal outcomes can be further reduced in this specific patient population of candidates for cerclage placement. Current evidence does suggest that if a woman has an indication for both 17-*α*OHPC prophylaxis as well as cerclage placement, it is reasonable to administer them concomitantly in the same pregnancy [13].

### 3.2. Reinforcing Cerclage

A literature search using the key words “repeat and cerclage,” “reinforcing and cerclage,” and “revision and cerclage,” demonstrated 22 citations and an additional 11 articles were found through other sources. Articles were excluded from this paper for the following reasons: 3 were review articles, 3 did not specifically report pregnancy outcome, 14 did not include patients with reinforcing cerclage placement during the study period, 4 articles focused on the topic of cerclage technique, 2 articles focused on cerclage complications such as infection and stromal erosion, 2 were case series of reinforcing cerclage without a reference group, and 1 publication was a study protocol without outcome results available. After exclusions, 4 remaining articles were included in this systematic review [14–17].

Women who undergo indicated cerclage placement and subsequently develop prolapse of the amniotic membranes to or past the level of the initial cervical cerclage may be considered “cerclage failures.” This can be identified by visual inspection or ultrasound examination after the cerclage placement. It has been suggested that these women may possibly benefit from a second or “reinforcing cerclage” in an attempt to delay delivery but there is a paucity of published data addressing the risks, benefits, and outcomes of this concept. The 4 studies included in this paper reported inconsistent findings of the incidence of early preterm birth and previable abortions with reinforcing cerclage (Table 2) [14–17]. Reinforcing cerclage placement when based on the finding of shortened cervical length on ultrasound was reported to be associated with a significantly increased risk for preterm birth and previable abortions [14]. Similar findings were reported in women with reinforcing cerclage after ultrasound evidence of prolapsing membranes [16]. Two studies reported a prolonged latency to delivery and live birth rate in women with reinforcing cerclage compared to a reference group of women without second cerclage placement following the finding of prolapsed membranes upon direct visualization [15, 17]. Although latency and live birth rates were increased in one study, all pregnancies ended in preterm birth <28 weeks gestation without the mention of neonatal outcomes [17]. A study by Fox et al. included all women with prolapsing membranes on physical exam who chose to have a reinforcing cerclage compared to women with no cervical change and no repeat cerclage. This study likely compares two different patient populations and outcome expectations, making meaningful conclusions challenging. 

 Based on the small body of evidence available, women are unlikely to have substantial benefit from a reinforcing cerclage when the choice to place it is based on ultrasound evidence of shortened cervix following initial cerclage placement. Women with “cerclage failure” who do not have evidence of infection, premature rupture of membranes, or labor may possibly have a longer latency period with a reinforcing cerclage as an emergency option; however, the decision to place the second cerclage should be exercised with caution and after thorough counseling of the patient regarding limited evidence to support its efficacy. Further relevant and well-performed studies are needed before reinforcing cerclage is routinely performed for this specific patient population, as the risk of preterm and previable birth is substantial. 

### 3.3. Prolonged Prophylactic or Empiric Tocolytic Therapy as an Adjunct to Cerclage

We performed a literature search using the keywords “atosiban and cerclage,” “beta mimetic and cerclage,” “terbutaline and cerclage,” “indomethacin and cerclage,” “magnesium and cerclage,” “nifedipine and cerclage,” and “calcium channel blocker and cerclage” with restriction to English language and human studies. These search criteria yielded 42 studies. Thirteen review articles were excluded, as were 5 articles on unrelated topics. Of the remaining 24 studies, 7 were excluded because they did not report the use of tocolytic therapies as an adjunct to a standard treatment of cerclage, 2 were excluded due to the use of transabdominal cerclage, 4 were excluded because they involved delayed interval delivery of a second twin, 3 due to both study groups receiving the tocolytic with no reference group of cerclage without a tocolytic for comparison, and 2 due to inconsistent reporting of specific tocolytic used in the study groups. A bibliographic review of the selected articles was also performed to search for additional studies which may have been missed with the original search terms yielding 2 additional articles. Of these 8 remaining articles, 3 case reports/case series were excluded as were 3 retrospective cohort studies in which both cerclage groups received tocolysis with no reference group of cerclage without tocolysis. This ultimately resulted in 2 retrospective cohort studies included in this systematic review comparing cerclage with tocolytic to a reference group of women who received cerclage without tocolytic (Table 3). 

We reviewed the 11 English language clinical trials included in the 2012 Cochrane review on cerclage [1] to determine the definition of “standard tocolytic therapy” as an adjunct to cerclage for the purpose of our systematic review paper. We defined no tocolytic treatment at the time of cerclage as the standard. 

Two retrospective cohort studies compared the use of indomethacin plus standard cerclage placement to a reference group of women who received cerclage without prophylactic indomethacin administration. Visintine retrospectively compared indomethacin use in a group of patients who received an ultrasound-indicated cerclage based on a cervical length <25 mm between 14 weeks and 23 weeks and 6 days [18]. The rate of spontaneous preterm birth prior to 35 weeks was similar between those who received indomethacin plus cerclage and those who received cerclage without indomethacin. A post hoc power analysis revealed that the study was underpowered to assess a benefit of indomethacin in addition to cerclage, if a benefit did exist. Berghella et al. published a retrospective cohort study of women with ≥1 cm cervical dilation at the cervical os between 14 weeks and 25 weeks and 6 days [19]. The patients were not randomly assigned to receive indomethacin, but rather the decision for indomethacin treatment was made at the discretion of the managing physician during the observational period studied. This study found no significant outcome differences between the cerclage group that received indomethacin versus the cerclage group that did not receive indomethacin. The reported risk for preterm birth <32 weeks and <35 weeks in women who received indomethacin suggested a possible benefit of indomethacin use but was not statistically significant with 95 percent confidence interval crossing the null value. However, women in this study who received indomethacin also had a higher rate of administration of steroids and antibiotics which could confound the reported outcomes in this group. A post hoc power analysis for this study also revealed that the study had insufficient sample size to show a statistically significant benefit of indomethacin plus cerclage versus cerclage alone, if a benefit did exist. These two studies suggest there may be benefit of indomethacin use with cerclage placement. However, based on the small sample size and observational nature of these studies, no definitive conclusions can be drawn regarding the efficacy of prophylactic indomethacin tocolysis proximate to the time of cerclage placement.

### 3.4. Antibiotic Use as an Adjunct to Cerclage

The correlation between subclinical infections or acute chorioamnionitis associated around the timing and/or after placement of cerclage has been established [20]. However, the efficacy of antibiotic use in cerclage candidates or recipients has not been extensively evaluated although it has been suggested as a needed adjunct by these findings. Prior to performing this literature review, we defined adjunctive antibiotic therapy as the use of any antibiotics for a treatment duration longer than 48 to 72 hours in the perioperative period [21], although the routine use of perioperative prophylactic antibiotics has not been established in history-indicated, ultrasound-indicated, or rescue cerclage procedures [13]. 

We performed a literature search using the key words “cerclage and antibiotics” restricted to the English language and human studies yielding 78 articles. Articles were excluded from this paper for the following reasons: 22 were literature review articles, 16 studies did not identify an inclusion group receiving only antibiotics and cerclage therapy, 6 did not specify antibiotic regimen or treatment duration, and 26 did not report the use of standard vaginal cerclage placement, but rather included other high-risk situations in which cerclage has been used (i.e., delayed interval delivery of twins, PPROM, documented infections, and “rescue” or “emergent” cerclage) and/or reported no outcome data in association with adjunct antibiotics. Seven remaining articles were excluded because they were unrelated to the topic of cerclage. No clinical trials or observational studies were found that compared pregnancy outcomes in women treated with cerclage plus adjunctive antibiotic therapy to a reference group that received cerclage only in the absence of other interventions (i.e., tocolytics and bedrest). We found that antibiotic treatment in cerclage recipients was inconsistently reported in the literature and often not clearly specified in clinical trial protocols regarding antibiotic class or treatment durations [22–24]. Therefore, our review yielded no studies that investigated the efficacy of antibiotics only as an adjunct to standard cervical cerclage placement. 

Of interest, one case series described the use of adjunctive antibiotic use in 10 women identified as having a history of failed cerclage in a prior pregnancy. These women were followed prospectively after ultrasound-indicated placement of cerclage between 14 and 24 weeks in the subsequent pregnancy and then administered an empiric prolonged antibiotic regimen of Ampicillin 250 mg orally daily for a month alternating with Erythromycin 250 mg orally daily for a month until delivery. The outcome was reported as prolongation of pregnancy in weeks, which was defined as the gestational age at time of delivery in current pregnancy minus the gestational age at delivery in the prior pregnancy. This study reported that the addition of continuous antibiotics as adjunctive therapy to cerclage in the second pregnancy did appear to improve length of gestation (mean increased latency of 13.4 weeks ± 4.2) in the setting of a prior failed cerclage [25]. However, no definite conclusion regarding the efficacy of antibiotics in cerclage patients can be determined from this case series given the absence of a reference group of women who received cerclage plus no antibiotic in the second pregnancy. The possibility that all of these women in their second pregnancy may have had prolonged latency compared to the prior pregnancy even in the absence of adjunctive antibiotics cannot be ruled out. 

In conclusion, there is insufficient evidence to support or discourage prolonged empiric antibiotic use as a sole adjunct to standard vaginal cerclage placement for the prevention of preterm birth. Randomized trials are needed to define a regimen and examine the potential efficacy of adjunctive antibiotic treatment following cerclage placement for the purpose of pregnancy prolongation or the prevention of preterm birth.

### 3.5. Bedrest as an Adjunct to Cerclage

We performed a literature search using the key words “bedrest and cerclage” restricted to the English language and human studies yielding 60 articles. Of those, 12 review articles were excluded as were 2 editorial/opinion articles and 4 case reports. We also excluded 5 articles whose focus were rescue or emergent cerclage, 2 questionnaire surveys, 18 articles on unrelated topics, and 17 studies that did not report the use of bedrest as an adjunct to a standard treatment of cerclage in comparison to a reference group of women who received cerclage without bedrest. Eleven studies were selected to review in detail as they specifically addressed the issue of cerclage and bedrest: five randomized controlled studies [21, 26–29], five prospective cohort studies [15, 17, 30–32], and one retrospective cohort study [33]. However, none of them specifically reported outcome differences of women who received cerclage with bedrest compared to women who received cerclage without activity restriction. Therefore, our paper yielded no comparative studies that specifically investigated the efficacy of bedrest as an adjunct to standard cervical cerclage placement. 

 During our review of the topic of bedrest used as an adjunct to cerclage, we found that a description of bedrest duration and a clear definition of specific activity limitations when employing bedrest for women with cerclage were inconsistently reported in the literature. A number of studies reported improved pregnancy outcomes with bedrest as an adjunct with cerclage compared to bedrest alone, but none specifically addressed cerclage with bedrest versus cerclage without bedrest. Most studies reviewed did not have a clearly presented definition of specific activity limitations assigned as bedrest and did not clearly describe bedrest as being prescribed as an inpatient or outpatient, and there was not a specifically stated gestational age in which it was discontinued. Therefore, we find insufficient evidence to support or refute the practice of bedrest used as an adjunctive therapy to cerclage placement in women at a high risk of preterm birth.

### 3.6. Vaginal Pessary as an Adjunct to Cerclage

A literature search using the key words “cerclage and pessary” restricted to the English language and human studies yielded 11 citations. Of those, 5 were review articles, 4 reported the use of vaginal pessary alone without cerclage and 2 reported the use of pessary with cerclage but did not include a reference group of women who received cerclage without pessary for comparison. We found no published observational studies or clinical trials reporting vaginal pessary used as an adjunct to standard cervical cerclage in comparison to cerclage alone. 

## 4. Conclusion

The use of vaginal cerclage as a preventive measure for spontaneous preterm birth in asymptomatic women has a growing body of evidence to support its efficacy in some subgroups of women such as women with singleton pregnancy who have had a prior preterm birth and have cervical shortening in the current pregnancy [1, 11]. With growing evidence to support cerclage use, questions grow more frequent regarding whether addition of other agents to cerclage may enhance its beneficial effects for pregnancy prolongation or significant reduction in adverse perinatal outcomes.

Other therapies have been reported to have beneficial effects for the reduction of preterm birth and improvement in perinatal outcomes when used as individual therapies in women at risk of spontaneous preterm birth without cerclage, such as progestins in the form of 17-*α*OHPC [12], vaginal progesterone [34, 35], indomethacin [36], and vaginal pessary [37]. Other interventions such as bedrest and empiric antibiotic administration are often prescribed to women at a high risk of preterm birth; however, there is no clear evidence to support their benefit for preterm birth prevention, and in fact they may be harmful [38–40]. The American College of Obstetricians and Gynecologists stated in the recent practice bulletin on the management of preterm labor (2012) that antibiotics should not be used to prolong gestation in women with preterm labor and intact membranes, and bedrest for the prevention of preterm birth should not be routinely recommended [41].

Through this systematic review, we found that no randomized trials have been published which were aimed to address the efficacy of any adjunctive therapy used with cerclage compared to a reference group of women who received cerclage alone. We identified several observational comparative studies on the topic of adjunctive therapies to cerclage through this systematic review; however, none clearly demonstrated a benefit over and above cerclage placement alone. The findings and conclusions of most of these studies are limited by small sample size and heterogeneity of treatments with concomitant use of multiple adjunctive therapies including bedrest, antibiotics, and/or tocolytics- often with inconsistent reporting of the use and duration of these interventions. 

We found that some therapies may have promise when used as an adjunctive treatment in addition to cerclage (indomethacin, 17-*α*OHPC) and others have not been studied (antibiotics, bedrest, and pessary). Similar therapies such as vaginal progesterone, nifedipine, magnesium sulfate, and other tocolytics have not also been evaluated in comparative studies as adjunctive treatment to cerclage compared to a reference group with cerclage alone. Based on available evidence, it is reasonable to consider the use of some agents such as 17-*α*OHPC or a 48-hour course of indomethacin in addition to cerclage, although definitive conclusions regarding their additive efficacy cannot be supported by the available evidence. Other therapies are unlikely to cause harm but have not been studied regarding efficacy when used in addition to cerclage (pessary and vaginal progesterone). Several others interventions have not been studied for evidence of efficacy when used with cerclage and may be potentially harmful (bedrest and empiric prolonged antibiotics), therefore we would not recommend that they are utilized as a routinely implemented adjunct to cerclage in asymptomatic patients until further studied in clinical trials. We encourage the development of future clinical trials with random allocation of individual adjunctive therapies plus cerclage compared to cerclage placement alone before their use in clinical practice is considered a standard of care.

## Figures and Tables

**Table 1:** Studies reporting the use of progesterone as an adjunct to cerclage.

Study	Design	Sample size	Documented average cervical length (mm)	Mean gestational age (weeks) cerclage	Cerclage type	Progesterone type	Total duration of progesterone therapy (weeks)	Gestational age (weeks) delivery	Outcome	Overall rate of PTB
Sherman 1966, [2]	Observational cohort*	33/540	n/a	Variable	Both	17-*α*OHPC IM	Variable to 36	39.3	LB	n/a

Vitoratos et al., 1996 [3]	Prospective cohort	8/18	n/a	14-15	Shirodkar	OHP IM^†^	Preoperative one dose	>37	PTB < 37 wga	6% (<37)^††^

Zakut and Lanciano, 1981 [4]	Retrospective cohort	30/60	14 ± 4.5	14.6 ± 2.83	Both	17-*α*OHPC IM	15	n/a	PTB < 24 wga hospitalization BW Apgar score	13.3% (<24)

Berghella et al., 2010 [5]	Retrospective cohort	47/148	19.0	18.9	McDonald	17-*α*OHPC IM	From 16 to 36	Not reported	PTB < 37 wga PTB < 35 wga PTB < 32 wga PTB < 28 wga PTB < 24 wga PM	49% (<37) 30% (<35) 17% (<32) 9% (<28) 4% (<24)

Rafael et al., 2011 [6]	Retrospective cohort	15/58	18 (12–22)	21 (16–22)	n/a	17-*α*OHPC IM	From 16–20 to 36	35 (27-28)	PTB < 35 wga PTB < 32 wga PTB < 28 wga BW	40% (<35) 33.3% (<32) 33.3% (<28)

Rebarber et al., 2008 [7]	Retrospective cohort	232/1882	n/a	n/a	n/a	17-*α*OHPC IM	13.5 ± 5	35.4 ± 4.7	PTB < 37 wga PTB < 35 wga PTB < 32 wga	40.5% (<37) 25.9% (<32) 13.4% (<32)

n/a: not available, 17-*α*OHPC: 17-alpha-hydroxyprogesterone acetate, IM: intramuscular, wga: weeks gestational age, PTB: preterm birth, LB: live birth, BW: birth weight, and PM: perinatal death (stillbirth or postnatal death prior to hospital discharge).

*Unable to specify based on the review of an article whether the observational study was prospective or retrospective in nature.

^†^This agent was reported as hydroxyprogesterone hexanoate.

^††^Did not specify whether PTB was in progesterone plus cerclage treatment group.

**Table 2:** Studies reporting the use of reinforcing cerclage.

Study	Design	Sample Size	Average cervical length at diagnosis (mm)	Cerclage type^‡^	Mean gestational age (weeks) initial cerclage	Mean gestational age (weeks) additional cerclage	Total number sutures placed	Mean latency to delivery (weeks)	Mean gestational age (weeks) at delivery	Outcome	Overall rate of PTB
Fox et al., 1998 [15]	Prospective cohort	12/26	n/a	McDonald	14*	20 (18–24)	2–4	13 (4–19)	34 (22–39)	PTB < 37 wga LB	75% (<37)
Baxter et al., 2005 [14]	Retrospective cohort	5/24	14.3 ± 3.6	McDonald	12^4/7^	19^1/7^	2	1^4/7^	20.8 ± 2.7	PTB < 35 wga PTB < 24 wga	100% (<35) 80% (<24)
Simcox and Shennan, 2012 [16]	Retrospective cohort	13/25	7.2 (0–15)	McDonald	14^5/7^	19^3/7^	2	6^4/7^	26^0/7^ ± 5 ^1/7^	PTB < 32 wga PTB < 24 wga	92% (<32) 38% (<24)
Song et al., 2011 [17]	Retrospective cohort	11/22	n/a	McDonald	17.0 ± 2.9	21.7 ± 2.3	2	5.1 ± 4.2	26.8 ± 5.0	LB BW	

n/a: not available, PTB: preterm birth, wga: weeks gestational age, LB: livebirth, and PM: perinatal mortality.

*Documented history-indicated cerclage was performed at 14 weeks gestation. Fifteen cases did not have documented gestational ages at initial cerclage placement.

**Table 3:** Studies reporting the use of empiric tocolytic agents as an adjunct to cerclage.

Study	Design	Sample Size	Cervical length cutoff for cerclage (mm)	Cerclage type^‡^	Mean gestational age (weeks)	Tocolytic	Duration	Outcome	Tocolytic group rate of PTB	Reference group rate of PTB
Visintine et al., 2008 [18]	Retrospective cohort	101	<25	Not specified	19 versus 19	Indomethacin	48 hours	PTB < 35 wga	39%	35%
Berghella et al., 2009 [19]	Retrospective cohort	222	Not specified	Not specified	19 versus 18	Indomethacin	48 hours	PTB < 35 wga PTB < 32 wga PTB < 28 wga	47% 39% 34%	58% 49% 37%

PTB: preterm birth and wga: weeks gestational age.
